# Palmitic Acid Induces Posttranslational Modifications of Tau Protein in Alzheimer’s Disease–Related Epitopes and Increases Intraneuronal Tau Levels

**DOI:** 10.1007/s12035-023-03886-8

**Published:** 2024-01-03

**Authors:** Valeria Melissa García-Cruz, Clorinda Arias

**Affiliations:** Departamento de Medicina Genómica y Toxicología Ambiental, Instituto de Investigaciones Biomédicas, Universidad Nacional Autónoma de México, Ciudad de México, CDMX 04510 México

**Keywords:** Palmitic acid, Tau phosphorylation, Tau acetylation, GSK3β, mTORC1, PP2A

## Abstract

Metabolic diseases derived from an unhealthy lifestyle have been linked with an increased risk for developing cognitive impairment and even Alzheimer’s disease (AD). Although high consumption of saturated fatty acids such as palmitic acid (PA) has been associated with the development of obesity and type II diabetes, the mechanisms connecting elevated neuronal PA levels and increased AD marker expression remain unclear. Among other effects, PA induces insulin resistance, increases intracellular calcium and reactive oxygen species (ROS) production, and reduces the NAD+/NADH ratio, resulting in decreased activity of the deacetylase Sirtuin1 (SIRT1) in neurons. These mechanisms may affect signaling pathways that impact the posttranslational modifications (PTMs) of the tau protein. To analyze the role played by PA in inducing the phosphorylation and acetylation of tau, we examined PTM changes in human tau in differentiated neurons from human neuroblastoma cells. We found changes in the phosphorylation state of several AD-related sites, namely, S199/202 and S214, that were mediated by a mechanism associated with the dysregulated activity of the kinases GSK3β and mTOR. PA also increased the acetylation of residue K280 and elevated total tau level after long exposure time. These findings provide information about the mechanisms by which saturated fatty acids cause tau PTMs that are similar to those observed in association with AD biochemical changes.

**Graphical Abstract:**

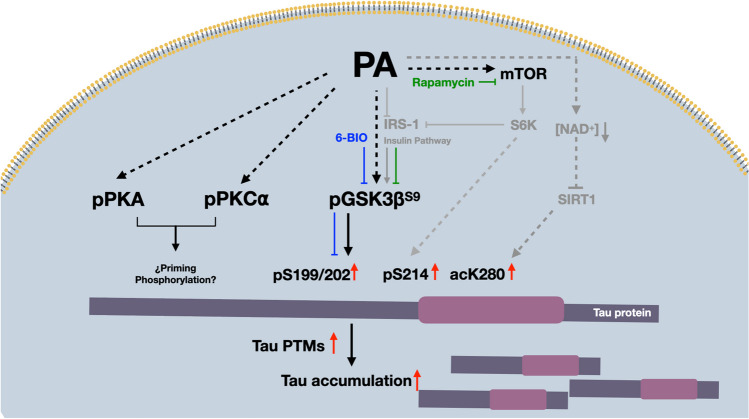

## Introduction

Alzheimer’s disease is the leading cause of dementia in elderly people and is characterized by the extracellular deposition of the amyloid-β peptide and intraneuronal accumulation of neurofibrillary tangles (NFTs) composed of hyperphosphorylated forms of the tau protein.

Tau is an intrinsically disordered protein that undergoes posttranslational modifications that regulate different cell functions but might also participate in the conversion of tau that leads to pathological states. Phosphorylation is the main PTM found in NFTs [1–3], but tau acetylation at specific residues has also been detected in the brains of AD patients [4]. Specifically, acetylation at K280 and K174 favors tau phosphorylation, diminishes tau degradation, and increases tau toxicity [5–10]. Tau PTMs may involve complex signaling pathways that alter the balance between the activity of protein kinases and protein phosphatases as well as acetylases and deacetylases.

Epidemiological studies have shown an important correlation between the presence of metabolic alterations and an increased risk for developing cognitive impairments. Several potentially modifiable risk factors for AD have recently been reported. Among the factors, obesity in midlife and diabetes in late life account for approximately 2% of all the risk factors attributable to sporadic AD [11]. These metabolic conditions have been largely associated with high consumption of lipids. Recent studies have reported increased concentrations of the saturated fatty acid palmitate (PA) in human cerebrospinal fluid from obese subjects compared with that in healthy humans [12, 13], suggesting that neurons may be exposed to high concentrations of PA in obese people. We found that neuronal exposure to PA induced insulin resistance, increased intracellular calcium and ROS production, and reduced the NAD+/NADH ratio, resulting in decreases in the content and activity of SIRT1 deacetylase [14]. All these processes may induce PTMs of tau through the dysregulation of protein kinase and deacetylase activities. However, the precise mechanism triggered by elevated PA on neurons and the association between PA and the metabolic disturbances leading to tau PTMs has not been elucidated. Therefore, the aim of the present work was to study the effects of PA exposure in differentiated neurons from human neuroblastoma cells, which were used as models to analyze human tau phosphorylation and acetylation at different AD-related epitopes (S199/202, S214, S356, S396, K280), and the protein kinases involved. We also studied the effects of these changes on the intraneuronal accumulation of tau.

## Material and Methods

### Cell Culture

**Fig. 1:**
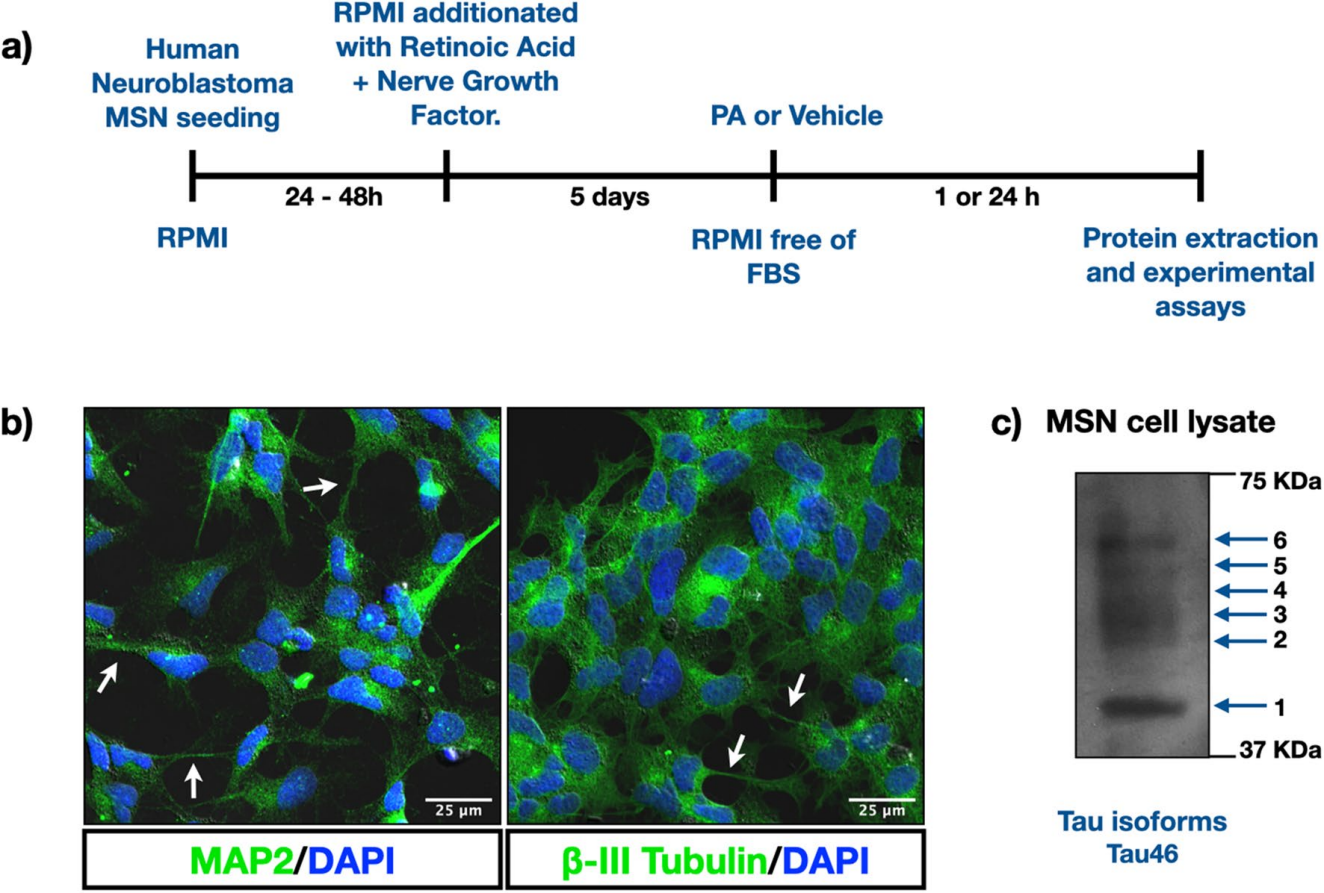
Experimental model and timeline. **a** Experimental protocol and timeline. **b** Confocal images of human MSN neuroblastoma cells after the differentiation protocol was completed. The differentiated neuroblastoma cells were stained with MAP2, β-III tubulin, and DAPI as a nuclear marker. White arrows indicate the presence of neurites. **c** Immunoprecipitation of tau protein from differentiated human neuroblastoma cells contains all six human isoforms

Human neuroblastoma cells (MSNs) (SMS-MSN cells, RRID:CVCL_7135) [15] were seeded at a density of 2.5 × 10^6^ cells per 35 mm well and maintained in RPMI 1640 medium containing nonessential amino acids plus 10% fetal bovine serum (FBS) (Gibco Invitrogen, Grand Island, CA) in an atmosphere with 5% CO_2_/95% O2 at 37 °C. This cell line is not among those listed by the International Cell Line Authentication Committee and was therefore authenticated, and ultimately, it was determined that the cell population was not contaminated with another human cell types. The cell lines were cultured for no more than 20 passages. Once they had adhered to plates, cells were driven to differentiate into a neuron-like phenotype by the replacement of the original medium with medium containing retinoic acid (10 μM) and nerve growth factor (NGF) (50 ng/mL) and subsequent culturing for 5 days. On the 5th day, the cultures were exposed to different treatments. The protocol to induce human neuroblastoma cell differentiation is similar to that reported by Encinas et al, 2000 [16] and yielded a neuronal population that exhibited many characteristics of mature neurons, such as long and extensively branched neurites and the expression of the neuronal markers, microtubule associated protein 2 (MAP2), and β-III tubulin. Immunofluorescence assays were carried out after the differentiation protocol was complete to demonstrate the acquisition of a neuron-like phenotype that produces all six human tau isoforms (Fig. 1).

### Immunofluorescence

Human neuroblastoma MSN cells were seeded at 150 × 10^3^ cells/well and differentiated on 12-microplates covered with coverslips. Once differentiation protocol was completed, cells were washed three times with cold-phosphate buffered saline (PBS) and fixed with paraformaldehyde (PFA) 1%/PBS for 5 min or with methanol for 10 min. Next, the cells were washed three times with PB 0.1M and permeabilized with 0.3% Triton X-100/PB 0.1M solution for 30 min at room temperature (RT). After incubation with blocking solution (bovine serum albumin (BSA) or 2%/PB 0.1M) for 2 h, the differentiated neuroblastoma cells were incubated with anti-MAP2 (1:1000, Millipore MAB378), anti-tubulin β-III (1:500 Sigma T2200), or anti-tau 46 (1:500, Cell Signaling #4019), antibodies overnight at 4 °C. Then, the cells were washed three times with 0.3% Triton X-100 PB 0.1M and incubated with Alexa Fluor 488 donkey anti-mouse or donkey anti-rabbit secondary antibody (1:1000 Thermo Fisher Scientific A-21202 and A21206, respectively) and Alexa Fluor 555 donkey anti-mouse (1:500 Thermo Fisher Scientific A-31570) for 2 h at RT. After two washes with 0.3% Triton X-100 PB 0.1M, nuclei were stained with 4′, 6-diamidino-2-fenilindol (DAPI) in PB 0.1M (1:5000 Roche Diagnostics) for 10 min and covered with fluorescent mounting medium DAKO (S3024). Confocal images were acquired with a Nikon confocal head coupled to an Eclipse Ti-E inverted microscope (Nikon Corporation, Tokio, Japan). Cells were analyzed under a PlanApo VC 60X WI DIC N2 (N.A. 1.2); single plane images were sequentially captured using standard galvanometric scanners, excitation wavelengths of 405 (0.64mW), 488 (0.43 mW), and 561 (3.5 mW) with AOTF modulation, pinhole aperture set at 14.05 um, and both standard and GaAsP detectors. Digital images were obtained with NIS-Elements C imaging software (Nikon) and processed with the ImageJ software program (Fig. 9). A 20X objective and digital 4X zoom are employed in Fig. 1.

### PA and Kinase Inhibitors Preparation and Treatment

PA (Sigma–Aldrich, USA) was prepared in an initial 200 mM stock solution using absolute ethanol (Sigma–Aldrich) as the diluent. From this stock solution, a 5 mM working solution was prepared on the day of the experiment by dilution using sterile PBS-BSA 10%. This solution was then incubated at 37 °C for at least 1 h with gentle shaking. Once the PA was completed diluted, it was added to the cultures at a final concentration of 200 μM. The chosen dose of PA is non-toxic for differentiated MSN cells, as we have previously shown [17] and is close to the PA concentration found in plasma from obese and diabetic patients [13, 18].

To maintain the fatty acid in solution, BSA was used as the carrier protein. The final concentration of ethanol in the culture medium was 0.1% (the experimental timeline is presented in Fig. 1a). The kinase antagonists, 6-bromo-indirubin-3′-oxime (6-BIO) and bisindolylmaleimide I (BIM-I), were purchased from Abcam (ab120891 and ab144264, respectively) and were prepared in a 1 mM stock solution in dimethyl sulfoxide (DMSO). A stock solution of rapamycin (Sigma R8781) prepared in DMSO (2.74 mM) and diluted with PBS to obtain a 100 μM solution. In all cases, inhibitors were added to the cultures 30 min before the PA at 1 μM (6-BIO and BIM-I) as well as 100 nM (rapamycin) in some experiments. The control conditions were established with vehicles used as diluents.

### Western Blotting

Human differentiated neuroblastoma cells were homogenized in RIPA lysis buffer (50 mM Tris pH 7.7, 0.1% SDS, 60 mM NaCl, 0.5% sodium deoxycholate, and 10% NP-40) and complete inhibitor cocktail from Roche Diagnostics at 4 °C. Protein concentration was determined using a Bio–Rad DC protein assay kit, and equal quantities of protein (80 μg for acetylated tau form and 25 μg for the rest of analyzed epitopes) were boiled in Laemmli buffer, separated by electrophoresis on acrylamide-SDS gel, and then transferred to a nitrocellulose membrane (Amersham, Germany No.10600016). Membranes were blocked with 5% bovine serum albumin-TBS-T or 5% nonfat dry milk-TBS-T pH 7 at RT for 2 h with gentle shaking. Then, the membranes were incubated overnight at 4 °C with the primary antibodies against tau 46 (1:500, Cell Signaling #4019), tau phosphorylated at S199/S202 (1:1000, Millipore AB9674), tau phosphorylated at S214 (1:1000, Abcam ab10891), tau p-S396 (1:1000, Abcam ab109390), tau p-S356 (1:1000, Abcam ab92682), tau acetylated at K280 (acetyl-K280; 1:1000, AnaSpec AS-56077), GSK3β phosphorylated at S9 (1:1000, Cell Signaling #9336), GSK3β (1:1000, Cell Signaling #9315), protein kinase C (PKCα) phosphorylated at S657 (ab180848), protein kinase A (PKA) α, β, ɣ phosphorylated at T197 (1:1000, Abcam ab75991), phospho-mTOR S2448 (1:1000, ThermoScientific #44-1125G), mTOR (1:1000, Cell Signaling #29725), and β-actin (1:1000, Sigma A5316). The membranes were washed 3 times with tris-buffered saline Tween (TBS-T) and incubated for 2 h at RT with the following secondary antibodies: horseradish peroxidase (HRP)-conjugated rabbit anti-mouse antibody (1:10 000, Invitrogen #61-6520) or goat anti-rabbit antibody (1:10 000, Invitrogen #31460). Then, the antibodies were visualized using a chemiluminescence EVL substrate (Millipore WBKLS0500) and Kodak X-Omat film. A densitometry analysis of the immunoblot was carried out using ImageJ software (NIH).

### Immunoprecipitation of tau Protein

Differentiated human neuroblastoma cells were lysed in RIPA buffer. A 500 μg of protein were incubated with protein A agarose beads (Millipore 16-125) and anti-tau 46 antibody (2 μg, Cell Signalling #4019). Total cell protein-bead-antibody mixture was incubated overnight at 4 °C in a rotating shaker. At the next day, the sample was centrifuge at 14,000 rpm for 5 min at 4 °C. Supernatant was discharged, and the pellet was washed 3 times with PBS. The precipitated proteins were boiled with loading buffer and analyzed by western blotting. The HRP-conjugate goat anti-mouse IgG Light-Chain (1:3000 Cell Signaling #91196S) was employed to detect total tau and to eliminate the possibility of detection of the heavy chains of the primary antibody.

### PP2A Immunoprecipitation and Phosphatase Activity Assay

A protein phosphatase 2A (PP2A) activity assay kit (Millipore, USA #17-313) was used in this experiment. Briefly, cultured differentiated neuroblastoma was treated with PA and was homogenized in low-concentration-phosphate buffer containing 20 mM imidazole-HCl, 2 mM ethylenediaminetetraacetic acid (EDTA), 2 mM ethylene glycol-bis (β-aminoethyl ether)-N, N, N′, N′-tetraacetic acid (EGTA) (pH 7.0), and complete inhibitor cocktail from Roche Diagnostics. The homogenates were centrifuged at 2000 × g for 5 min at 4 °C. Supernatants were collected, and a volume containing 100 μg of protein was incubated with the anti-PP2A C subunit and protein A agarose beads for 2 h at 4 °C in a shaker. The beads were washed 3 times with TBS and washed one with the Ser/Thr assay buffer provided in the kit. Sixty microliters (final concentration of 750 μM) of diluted phosphopeptide (K-R-pT-I-R-R) were added and incubated with the cells for 10 min at 30 °C with gentle shaking. Then, PP2A activity was measured using malachite green phosphate detection buffer, and after developing color (15 min), the samples were analyzed at 630 nm with a spectrophotometer. The results are presented as the percentage of activity relative to controls.

### Statistical Analysis

All data are expressed as the mean ± standard error (SEM). Comparisons among control and each treatment were made using two-tailed Student’s *t* tests. Differences were considered statistically significant when *p* < 0.05. GraphPad Prism 8.0 (GraphPad Software Inc.) was used for generating dot plot graphs and for statistical analyses. The results are expressed as a percentage relative to the control group from each individual experiment. Control data are depicted as the percentage of the mean of all control experiments for each experimental condition.

## Results

### Differential Changes in tau Phosphoepitopes after PA Treatment

**Fig. 2:**
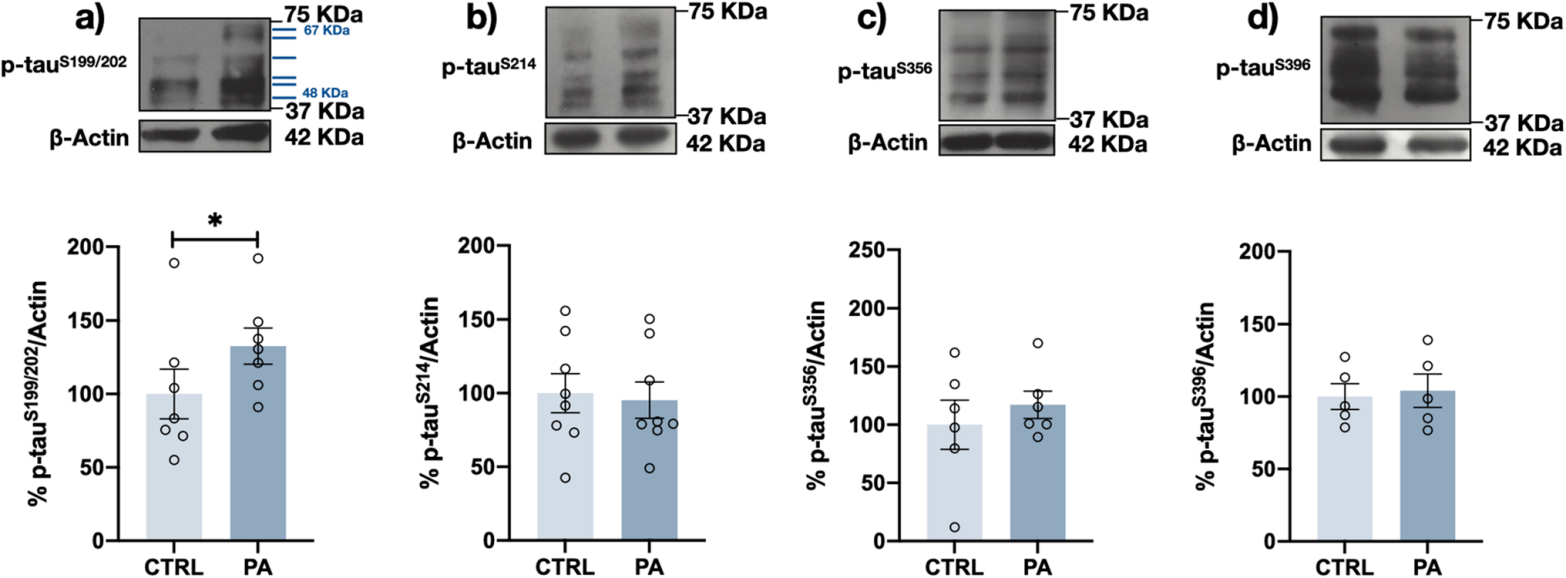
PA increased S199/S202 tau phosphorylation after 1 h. Representative Western blots and densitometry analysis results for 6 human tau isoforms in which different phosphoepitopes were stained: **a** p-S199/S202, **b** p-S214, **c** p-S356, and **d** p-S396. After 1 h of neuronal exposure to 200 μM PA, an increase in p-S199/S202 was found. Graph bars represent the mean ± SEM from at least 5–8 independent experiments; *p < 0.05 (Student’s t test)

**Fig. 3:**
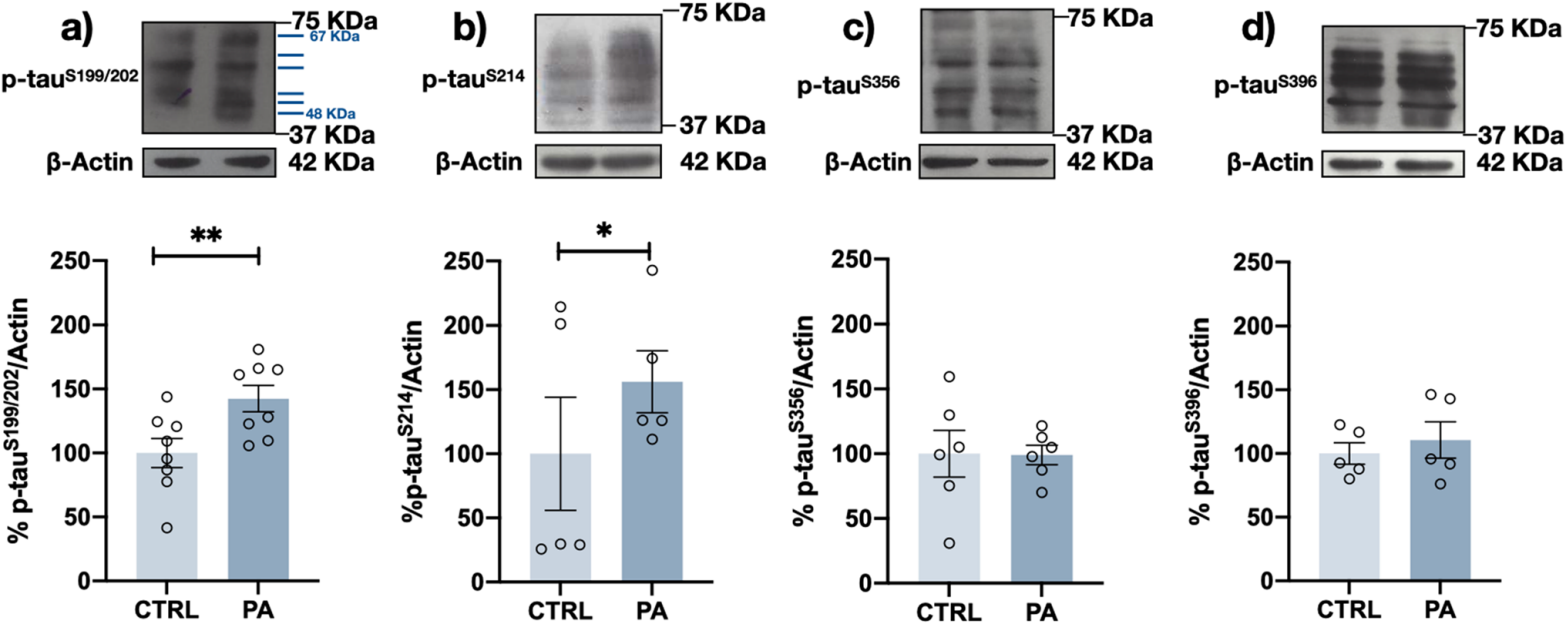
PA induced increases in S199/S202 and S214 tau phosphorylation after 24 h. Representative Western blots and densitometry analysis results for 6 human tau isoforms in which different phosphoepitopes were stained: **a** p-S199/S202, **b** p-S214, **c** p-S356, and **d** p-S396. Differentiated human neuroblastoma cells were exposed to 200 μM PA for 24 h. A significant increase in S199/S202 and S214 phosphorylation was observed. Graph bars represent the mean ± SEM from at least 5–8 independent experiments; *p < 0.05, **p < 0.01 (Student’s *t* test)

Since PA leads to changes in signaling pathways that may induce the activation of different kinases, we first examined the phosphorylation state of tau at four different phosphoepitopes (S199/S202, S214, S356, and S396), because the phosphorylation levels at these sites have been previously found to be increased in the brains of AD patients. Differentiated human MSN cells were incubated with PA for 1 or 24 h, and the level of tau phosphorylation was measured. After 1 h of PA incubation, a significant increase in tau phosphorylation was detected only at p-S199/S202 (30% vs. control) (Fig. 2a). The phosphorylation of this epitope was still evident (a 40% increase vs. the control) after 24 h of PA treatment (Fig. 3a). The phosphorylation at the S214 site was also increased by approximately 50% (Fig. 3b) after 24 h of PA exposure, while the level of phosphorylation at the other two phosphorylation sites, S356 and S396, did not change regardless of the duration of PA exposure (Figs. 2 c and d and 3c and d).

### Protein Kinases Involved in tau Phosphorylation after PA Exposure

**Fig. 4:**
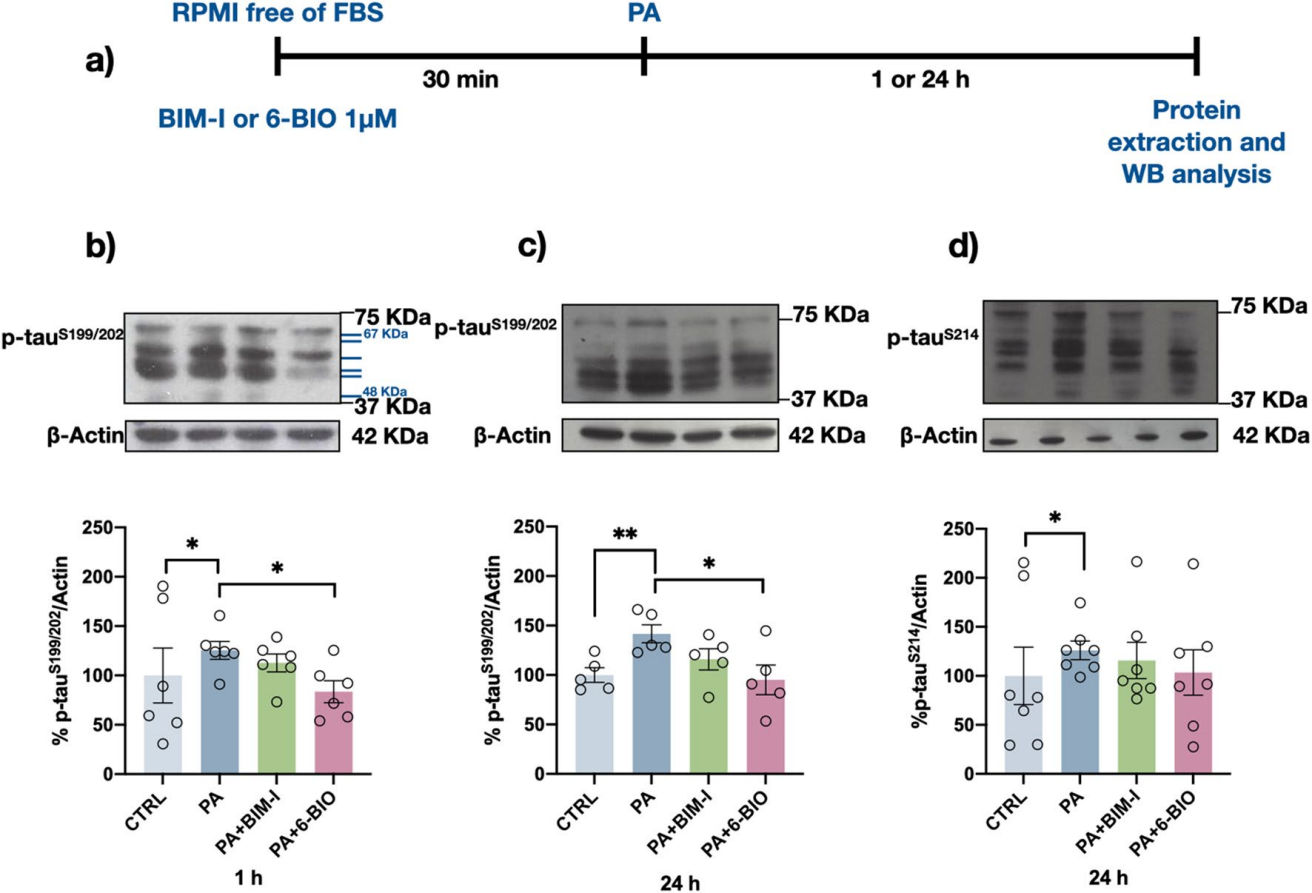
PA-induced phosphorylation of S199/S202 is mediated by GSK3β. Experimental design and timeline of the experiment (**a**). Representative Western blots and densitometry analysis results showing the levels of p-tauS199/S202 (**b**, **c**) and p-tauS214 (**d**) after GSK3β and cPKC inhibition. Differentiated human neuroblastoma cells were exposed for 1 h (**b**) or 24 h (**c**, **d**) to 200 μM PA in the absence or presence of BIM-I (1 μM) or 6-BIO (1 μM). Hyperphosphorylation of S199/S202 was blocked by the GSK3β inhibitor 6-BIO, while cPKC inhibition by BIM-1 did not change the phosphorylation level of any epitope at any of the times it was measured. Graph bars represent the mean ± SEM from 5–7 independent experiments; *p < 0.05, **p < 0.01 (Student’s *t* test)

Because we have previously reported that PA causes insulin resistance in neurons [17] and mobilizes external Ca^2+^ [19], we wanted to study the involvement of the kinases GSK3β and PKCα in the phosphorylation of the two tau residues that we found to exhibit upregulated phosphorylation. As shown in Fig. 4, the level of p-S199/S202 (Fig. 4b, c) at the two times it was measured was significantly decreased in the presence of the GSK3β inhibitor 6-BIO, while the level of p-S214 was unaffected (Fig. 4d).

**Fig. 5:**
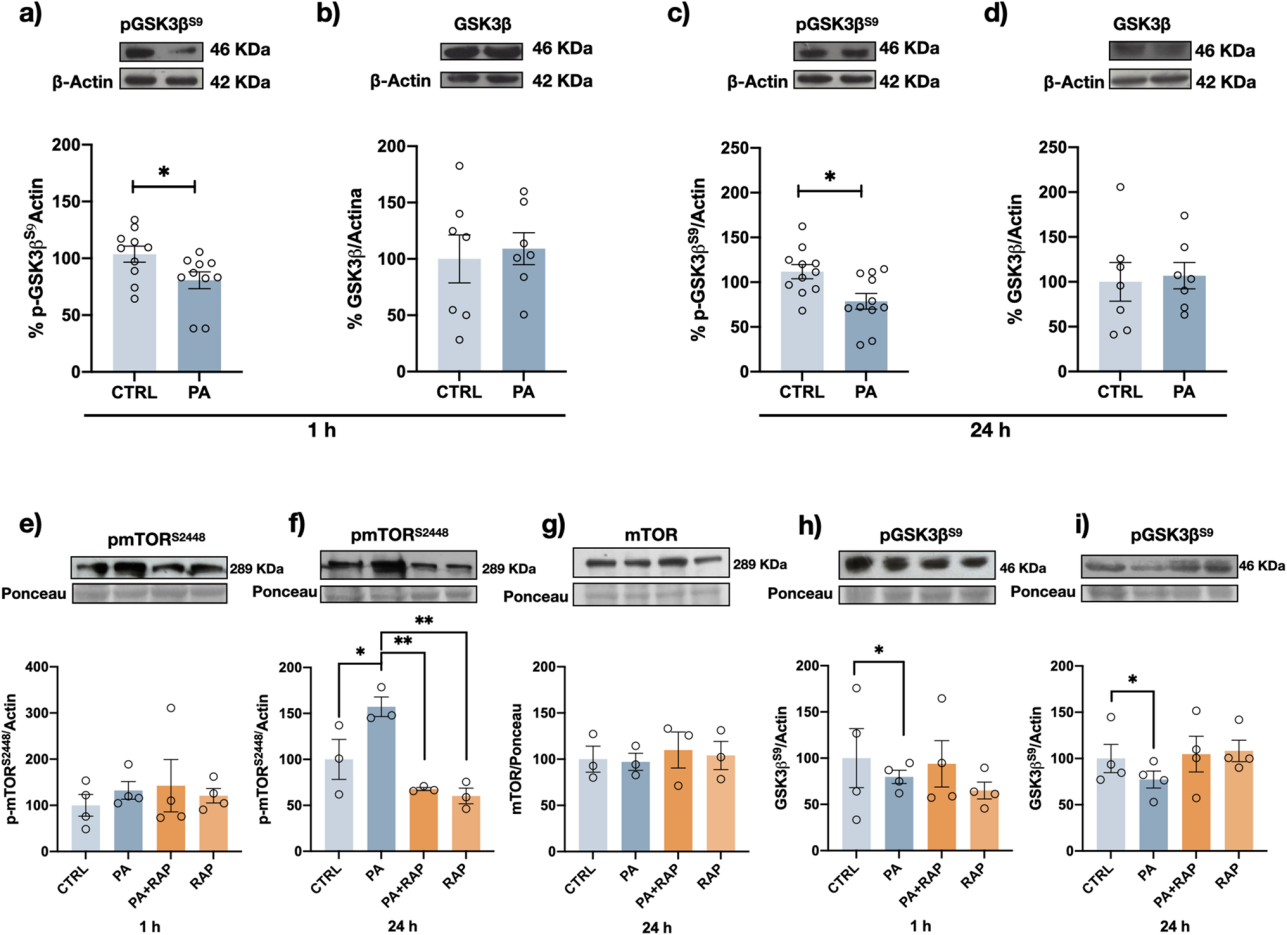
PA upregulates GSK3β activity mediated via mTORC1. Representative Western blots and densitometry analysis results showing the p-S9GSK3β and total GSK3β levels after 200 μM PA exposure for 1 h (**a**, **b**) or 24 h (**c**, **d**). The p-S9GSK3β content was reduced after PA treatment, but no change in the total level of the protein was observed. The p-mTOR^S2248^ levels after 1 h (**e**) and 24 h (**f**) of PA were enhanced and prevented by rapamycin at 24 h treatment without changes of total mTOR levels after 24 h of PA (**g**). PA-induced reduction of p-S9GSK3β was blocked after 1 (**h**) and 24 h (**i**) of rapamycin. Graph bars represent the mean ± SEM from at least 3-11 independent experiments; *p < 0.05, **p < 0.01 (Student’s *t* test)

**Fig. 6:**
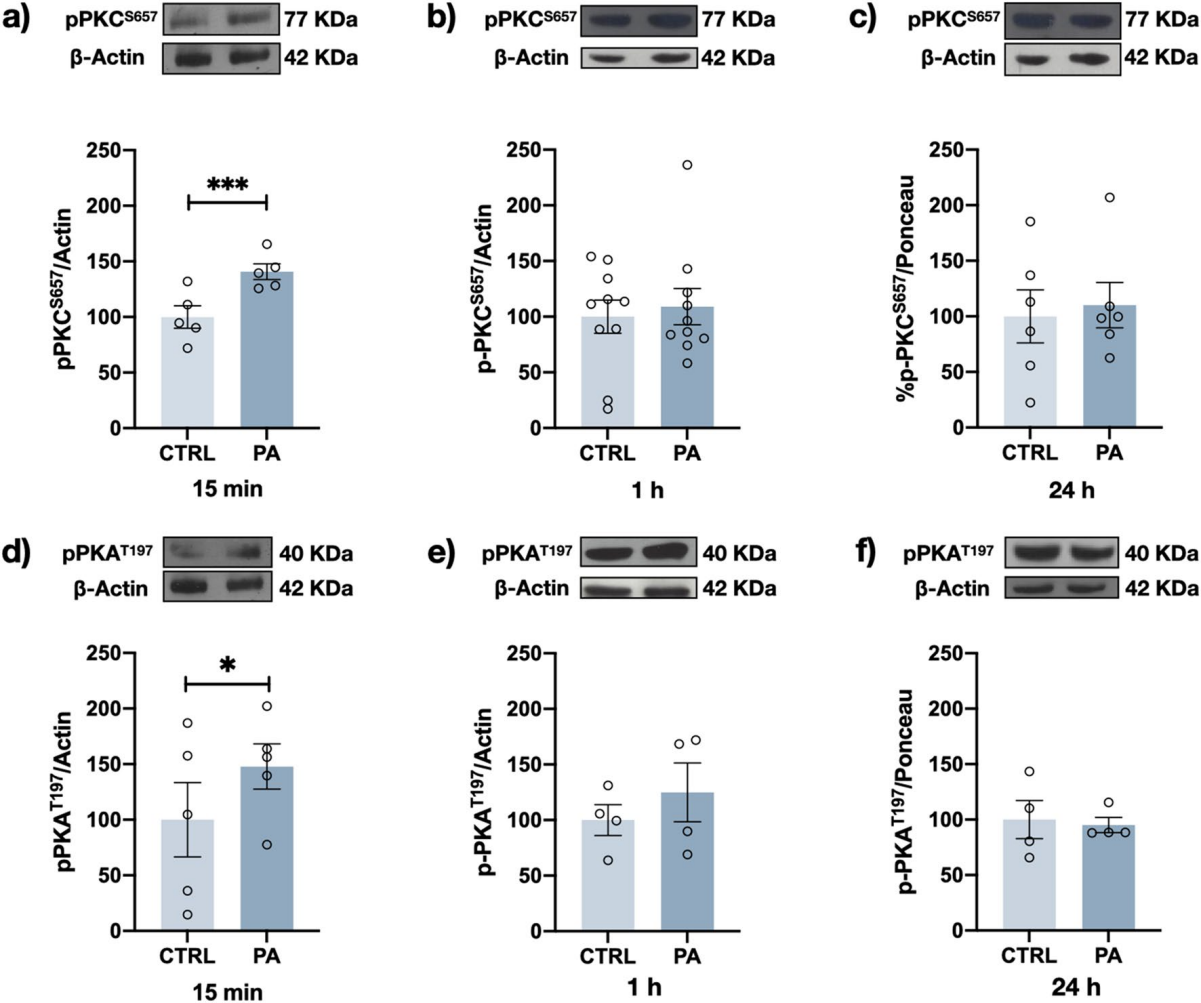
PA induces the transient activation of PKC⍺ and PKA. Representative Western blots and densitometry analysis results for p-S657PKC⍺ (**a**, **b**, **c**) and p-T197PKA (**d**, **e**, **f**) after 200 μM PA exposure for 15 min (**a**, **d**), 1 h (**b**, **e**), and 24 h (**c**, **f**). Graph bars represent the mean ± SEM from at least 4–10 independent experiments; *p < 0.05, ***p < 0.005 (Student’s t test)

To determine whether the activity of different kinases is affected by PA exposure, we measured changes in the phosphorylation level of specific residues in GSK3β, PKCα, and PKA, which would reflect protein activation. For GSK3β, we compared the levels of S9 phosphorylation in control cells and in cells exposed for 1 and 24 h to PA (Fig. 5). As measured at both times, PA produced a significant decrease in the level of the inhibitory residue p-S9, demonstrating the activation of the enzyme (Fig. 5a, c) without changes in the total content of the enzyme GSK3β (Fig. 5b, d). To further investigate the mechanism involved in PA-induced GSK3β activation under the condition in which the insulin-dependent activation of the phosphatidyl inositol 3-kinase (PI3K)/Akt pathway was absent, we analyzed whether PA upregulates the activity of the kinase mTORC1. When cells were exposed to PA, the amount of the active form of mTORC1 (p-S2448) was increased starting at 1 h of PA incubation, showing a statistically significant after 24 h (50% vs. control) (Fig. 5e, f) without change in the total protein content (Fig. 5g). As expected, in the presence of the mTOR inhibitor rapamycin, the PA-dependent activation of GSK3β (reduction of p-S9) was completely blocked, corroborating that the PA-dependent activation of GSK3β is mediated via mTORC1 (Fig. 5h, i). PA treatment also produced a transient increase in the phosphorylation level of residue S657 in PKCα after 15 min of exposure, which was diminished 1 h and 24 h later (Fig. 6a–c). A similar effect was observed with the kinase PKA, which was transiently activated after 15 min of PA treatment (Fig. 6d–f).

### PA Increases tau Acetylation was Accompanied by Intraneuronal tau Accumulation

**Fig. 7:**
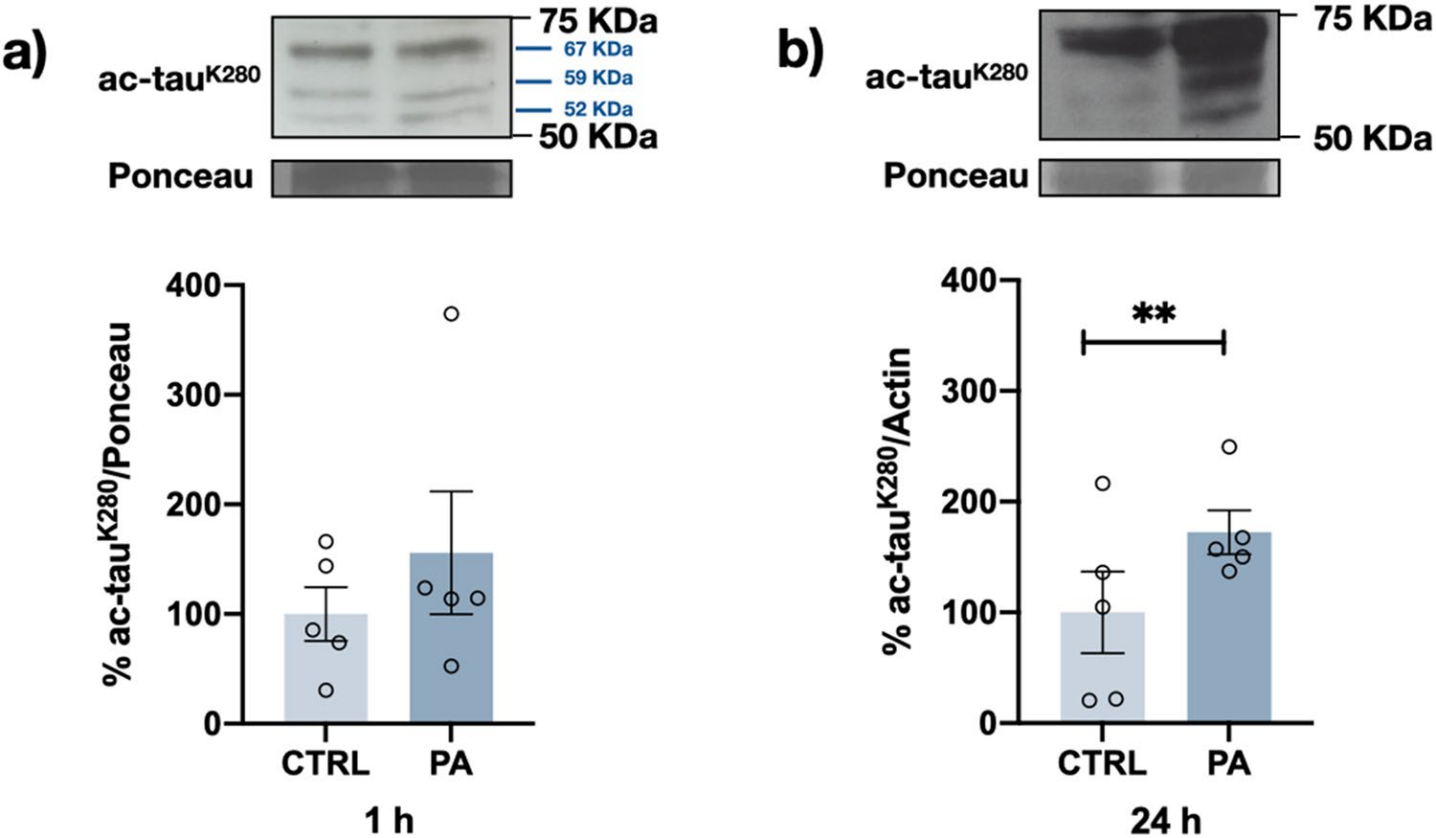
PA induces tau acetylation. Representative western blot and densitometry analysis results showing ac-K280tau after 200 μM PA treatment for 1 h (**a**) and 24 h (**b**). A significant increase in tau acetylation was evident after 24 h of PA treatment. Graph bars represent the mean percentages ± SEM from 5 independent experiments; **p < 0.01 (Student’s *t* test)

Concomitant with the observed changes in tau phosphorylation, differentiated neuroblastoma cells exposed to PA also showed increased tau acetylation at the K280 residue. Although a slight trend showing increased tau acetylation was observed during the first hour of PA exposure (Fig. 7a), this effect was statistically significant at 24 h, when it was than twofold higher than that of the control (Fig. 7b). As shown in this figure, acetylation at K280 was observed only in the three isoforms carrying four repeats of tau with this lysine residue.

**Fig. 8:**
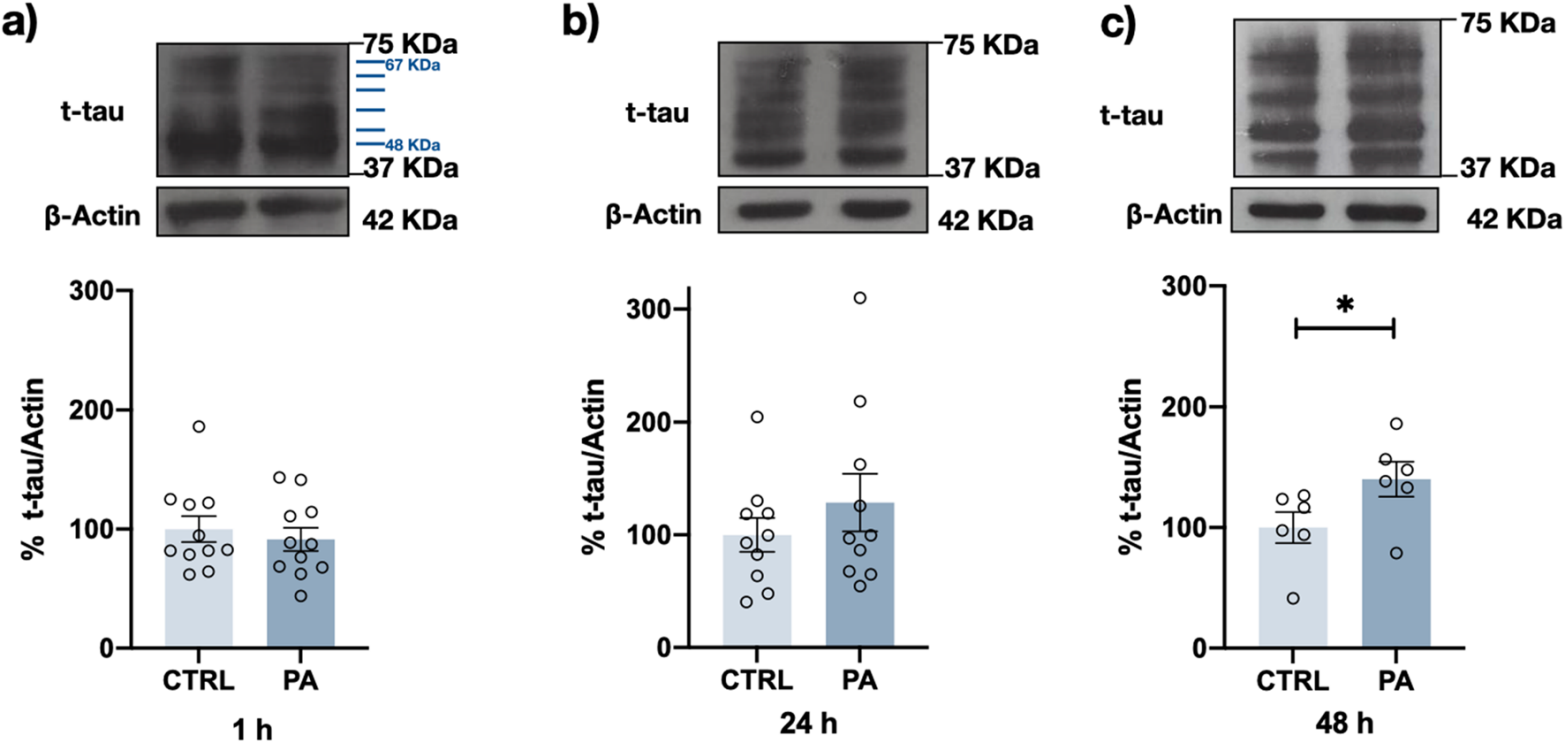
PA increases the total tau content. Representative Western blots and densitometry analysis results showing the total tau level after 1 h (**a**) and 24 h (**b**) or 48 h (**c**) 200 μM PA exposure. Total tau content was significantly increased after 48 h of PA treatment. Graph bars represent the mean ± SEM from 6 to 11 independent experiments; *p < 0.05 (Student’s *t* test)

To analyze whether the PA-induced PTMs of tau exert an impact on intraneuronal tau accumulation, we measured total tau in neurons exposed to PA for 1, 24, and 48 h. Western blot analysis confirmed a 40% increase in the intraneuronal tau content after 48 h of PA treatment (Fig. 8a–c).

### Changes in tau Distribution after PA

**Fig. 9:**
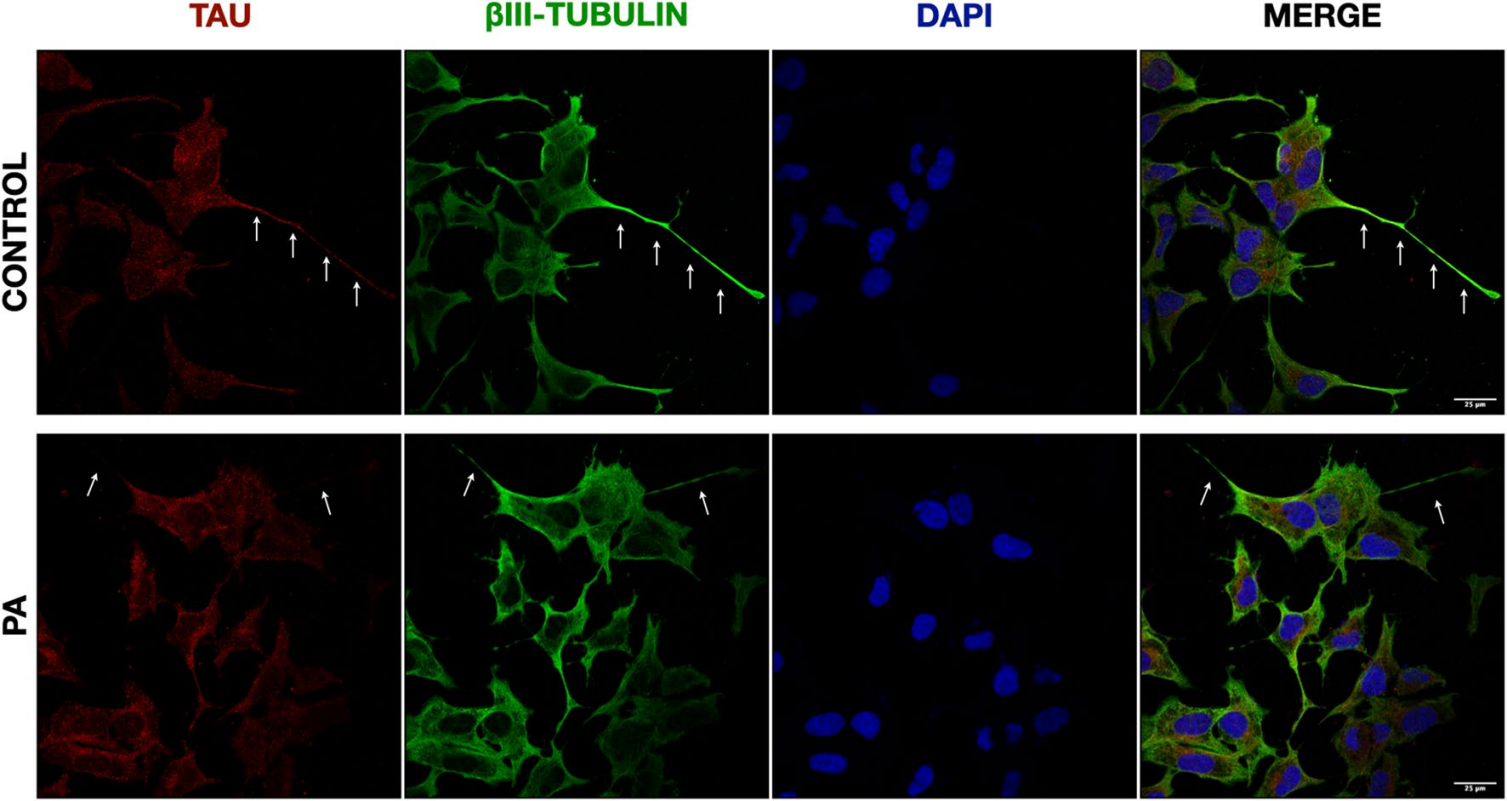
Tau protein distribution in differentiated neuroblastoma cells after incubation with PA. Confocal images of differentiated MSN neurons before and after 24 h of exposure to 200 μM PA. Cells were stained with β-III tubulin (green), tau-46 (red), and nuclei with DAPI (blue). Arrows indicate long extended neurites stained with tau. Images at 60X are showed. Scale bars: 25 μm. Arrows represent elongated neurites with high concentration of tau under control condition compared to shorter neurites with reduced tau content after PA

Intraneuronal localization of tau was determined by immunofluorescence in differentiated neuroblastoma cells before and after PA treatment. Under basal conditions, tau was found abundantly distributed in the soma and processes of the neuron-like cells. After 24 h of PA exposure, tau distribution showed a disorganized pattern with reduced content in neurites that appeared shorter although without observable changes in total protein contents (Fig. 9).

### PA-induced Changes in Protein Phosphatase 2A Activity

**Fig. 10:**
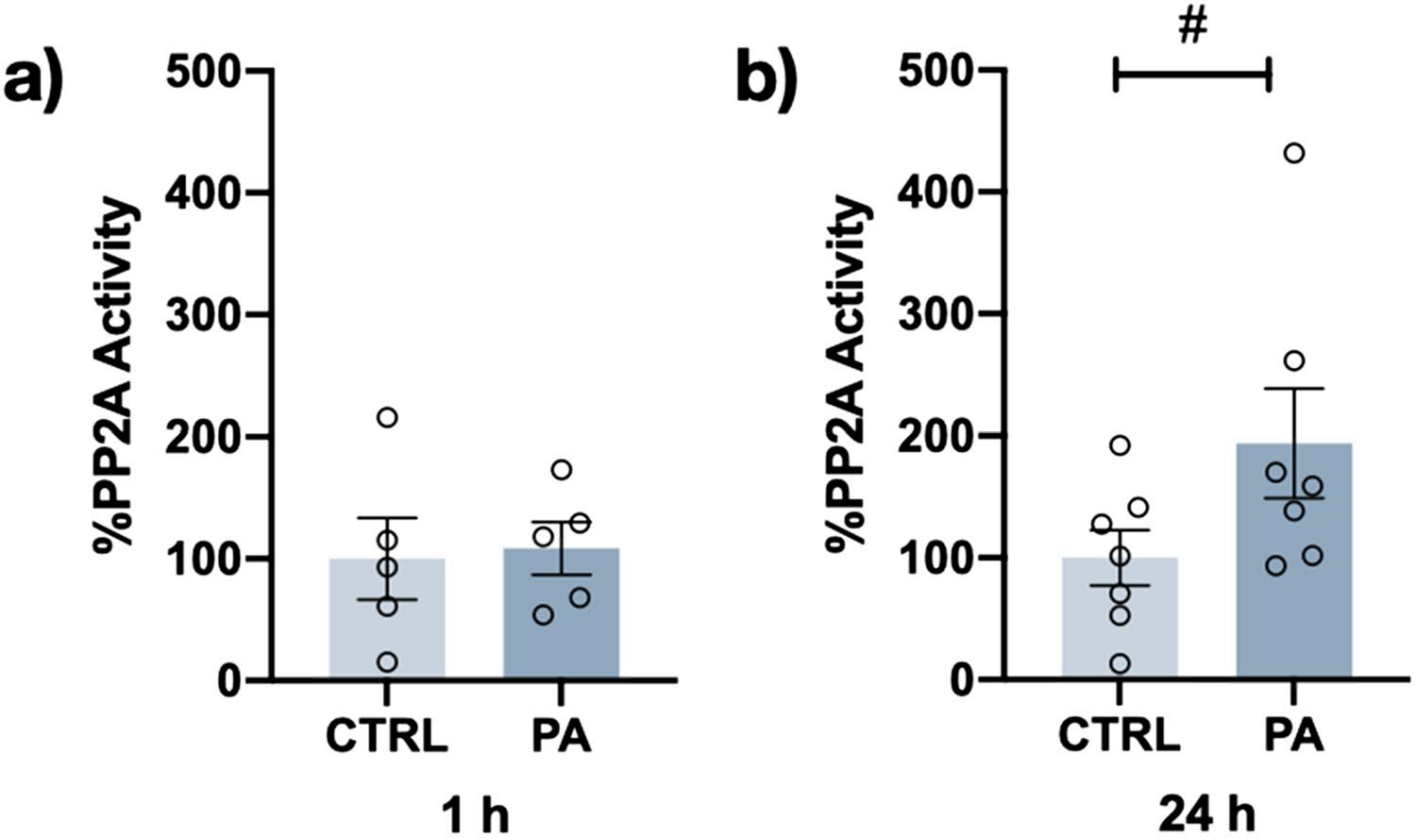
Effect of PA on PP2A activity. Green malachite assay was used to determine the activity of the catalytic subunit of PP2A after 1 (**a**) or 24 h (**b**) of 200 μM PA treatment. A trend culminating in a twofold increase in PP2A activation was observed after 24 h of PA treatment. Data are expressed as percentage of activity. Graph bars represent the mean ± SEM from 5 to 7 independent experiments; *#p* = 0.0584 (Student’s *t* test)

Because the metabolic pathways followed by PA treatment in cells lead to the formation of ceramides, which can activate the protein phosphatase 2A (PP2A) [20], we wanted to determine whether the activity level of PP2A was increased in cells treated with PA. The activity of PP2A, one of the main protein phosphatases involved in tau dephosphorylation (Fig. 10), was not changed after 1 h of PA exposure (Figure 10a), but a significant increase in PP2A activity, up to a twofold increase (#*p* = 0.0584), was found after 24 h of PA treatment (Fig. 10b).

## Discussion

The intake of a high fat diet has been associated with the development of metabolic diseases and the onset of neurodegenerative diseases [21–24]. The main component of this type of diet is the saturated fatty acid PA, the metabolism of which leads to a variety of consequences in neurons. In this study, we provided evidence showing how neuronal exposure to high but not toxic concentrations of the saturated fatty acid PA induced the PTMs of human tau. The role of phosphorylation and acetylation of tau in the brains of patients with AD has been established, but no clearcut evidence showing the molecular mechanisms that link metabolic risk factors with biochemical changes in tau had been provided to date. Herein, we demonstrated that exposure to PA activated signaling cascades that exerted impacts on the activity of the main kinases involved in tau phosphorylation at specific residues and increased tau acetylation, resulting in intraneuronal tau accumulation.

Neuronal exposure to PA induced biochemical modifications of tau via changes in different phosphoepitopes. The most affected site was S199/S202, in which phosphorylation was increased as early as 1 h and remained elevated 24 h after PA treatment. This site is a site for GSK3β and PKA phosphorylation [25–27]. In fact, we found that the phosphorylation of this residue induced by PA depends upon the activation of GSK3β, as indicated by the effect of the specific GSK3β inhibitor, 6-BIO blunting its phosphorylation, and because PA induced a reduction in the inhibitory residue p-S9 in this kinase. Interestingly, p-S199/S202 has been found to be elevated in the brains of AD patients and has also been reported to be hyperphosphorylated in the pretangle stages of NFT formation [28]. GSK3β is considered to be one of the main kinases that phosphorylates tau in vivo [29–31], and in this study, we found that PA induced the activation of this enzyme, possibly mediated by an inhibitory mechanism exerted by PA on the insulin/PI3K/Akt pathway, as shown in several works with cultured neurons performed by our group and other teams [17, 19, 32–34]. In contrast, in the present study, the effects of PA on cells were examined in the absence of insulin-producing stimuli; that is, we analyzed the possible inhibition of the PI3K/Akt pathway downstream of insulin. As expected, we found that mTORC1 kinase, which is a nutrient sensor and metabolic regulator, was activated by PA treatment, similar to the effect reported in hepatocytes and skeletal muscle cells [35, 36]. Moreover, PA-dependent GSK3β activation was completely blocked by the mTOR inhibitor rapamycin. Activation of mTORC1 promotes downregulation of insulin signaling through insulin receptor substrate-1 (IRS-1) phosphorylation at serine residues by the ribosomal serine/threonine 6 kinase (S6K), which is an mTOR effector molecule [37–39] resulting in GSK3β activation.

S199/S202 is a tau site that is also a substrate of PKA, which we found transiently activated 15 min, as well as PKC⍺, after PA exposure. The activation of PKCα did not seem to be involved in PA-mediated tau phosphorylation because this phosphorylation event was not inhibited by BIM-1. According to previous reports, PKA-dependent tau phosphorylation might prime tau to facilitate further GSK3β phosphorylation, a phenomenon that may have occurred in the present study [40–42]. The mechanism underlying PKA activation after PA exposure is not completely clear, but there is evidence suggesting that orphan G protein-coupled receptor 40 (GPR40) is activated by long-chain saturated fatty acids, leading to increased cAMP levels and PKA activation [43].

We have also found the phosphorylation of S214 in differentiated neuroblastoma cells exposed to PA but only after the longer exposure time (24 h), which suggests that other mechanisms also participate in the modification of this phosphoepitope, since the pharmacological inhibitors of GSK3β and PKCα did not reduce the phosphorylation of this site. Among these mechanisms, the involvement of S6K is possible because this kinase is the target of mTORC1 and has been previously shown to phosphorylate tau S214 [44–46]. This phosphoresidue is important since it has been found to be hyperphosphorylated in PHFs, which eventually leads to larger aggregates of tau protein [47]. It is important to note that the increase in tau phosphorylation was not associated with changes in the total protein contents at 1 or 24 h after PA treatment, but cellular localization revealed a disorganized distribution pattern. PA did not exert general detectable effect on tau phosphorylation, as evidence by the phosphorylation of S356 and S396 remaining unchanged. The p-S356 epitope is situated in the microtubule repeat domain region and is highly phosphorylated in PHFs in AD patients, thus participating in the detachment of tau from microtubules and reducing its microtubule affinity. Moreover, site S356 is a common target of AMPK [48–51]. The p-S396 (also known as the PHF1 site) is located in the C-terminus of the tau protein and has been identified in AD brains at late stages of pathology [28, 52]. Although this residue can also be phosphorylated by GSK3β, we found that it was highly phosphorylated under basal conditions, so it would be possible that additional phosphorylation could not be detected after PA stimulation.

The role of PA-induced GSK3β activation in the phosphorylation of various tau residues may be expanded to other taupathies characterized by aggregation of hyperphosphorylated tau. In fact, some inhibitors of this kinase are being tested in clinical trials for the treatment of Progressive Supranuclear Palsy and Frontotemporal Dementia [53].

In addition to the effects of PA on tau phosphorylation, long-term PA exposure led to a trend toward increased PP2A activity. This effect may represent a cellular response counteracting tau phosphorylation but may also directly depend on the PA downstream metabolite ceramide, which is a PP2A activator [54]. PP2A may also impair PI3K/Akt signaling by dephosphorylating Akt [55, 56]. PP2A is one of the main tau phosphatases [57, 58], but it has been reported that phosphorylation in the proline-rich region of tau harboring S199/S202 and S214 [59, 60] can impede the interaction between tau and PP2A [61–63], which may explain the maintenance of the hyperphosphorylated state of the protein despite apparent PP2A activation.

Another PTM analyzed in the present study was PA-dependent tau acetylation at K280, which has been found to be elevated in the brains of patients with AD or other tauopathies [64] to promote pathological tau aggregation [5]. The mechanism underlying this association is not well known but suggests an imbalance between the activities of acetylases and deacetylases. The NAD+-dependent class-III protein deacetylase SIRT1 is one of the major enzymes involved in the removal of acetyl groups from tau in vitro and in vivo [65]. Studying PA-treated hippocampal neurons, we previously reported a reduction in SIRT1 activity because of reduced NAD+ availability [14]. Tau acetylation at specific residues can affect the tau phosphorylation profile [9] revealing complex relationships between different PTMs to transform tau into a pathological molecule. The PTMs induced by PA were associated with intraneuronal tau accumulation at longer incubation time (48 h), which may reflect insufficient clearance of the modified proteins. Tau acetylation at some residues impedes the recognition for ubiquitination [66] and inhibits chaperone-mediated autophagy, contributing to AD progression [8]. However, the increase in total tau that was observed after 48 h of PA could also be attributed to an increase in tau expression.

In summary, we provide evidence showing the possible mechanisms by which PA treatment exerts an impact on neuronal metabolism, leading to the PTMs and accumulation of the tau protein. These mechanisms may contribute to the pathological changes in tau associated with increased saturated fatty acid availability that is caused by high intake of saturated fat.

## Data Availability

Datasets generated in the present study are available on request to the corresponding author.
